# Supplementary material for: Identification of Freezing-Responsive microRNAs and Their Targets in Chinese Jujube by Small RNA and Degradome Sequencing
Source: Int J Mol Sci. 2026 Sep 4;27(17):7912. doi: 10.3390/ijms27177912 (PMC13566890; doi:10.3390/ijms27177912)
Supplement: Supplementary file 1 [file ijms-27-07912-s001.zip › Supplementary Material S2/novel_miR_113.pdf]

Provisional ID : novel_miR_113
Score total : 201.2
Score for star read(s) : 3.9
Score for read counts : 199.5
Score for mfe : 0
Score for randfold : -2.2
Score for cons. seed :
Total read count : 403
Mature read count : 401
Loop read count : 1
Star read count : 1

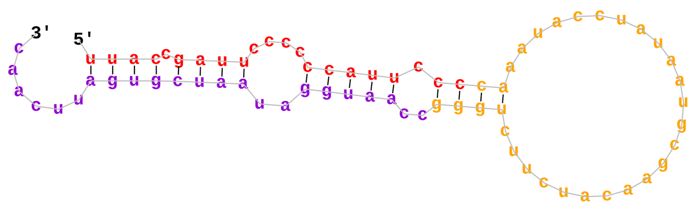

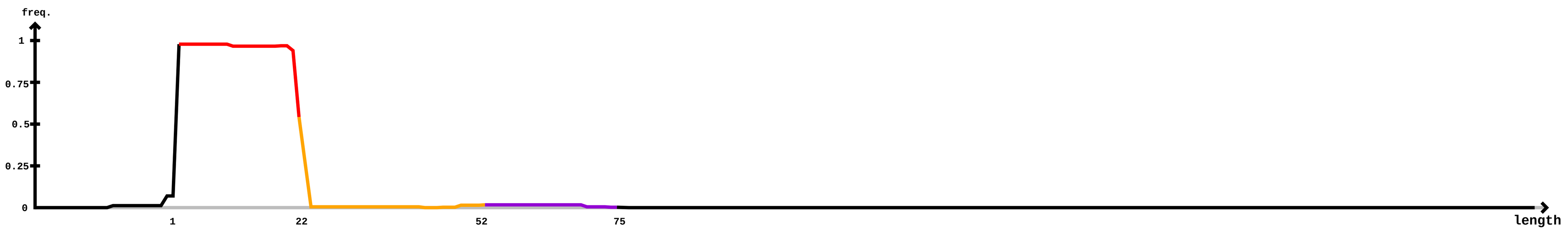

Mature Star

```
5'- aacaaugguagcaagggugguuuuaccgauuccccccauuccccaaauaccuauaaugcgaacaucuucugggccaauggauaaucgugauucaacauccuuaauauuuucuuuaauuccaaaaagcaacuccuuugaaugcucauauguugaucgguauuuaggcaauuucucuaaaauaucuuuaacaacucguuuaacuaacucauacucaggccuacaaaaaauaaauaaauaaauauuguuagac -3' obs
    aacaaugguagcaagggugguuuuaccgauuccccccauuccccaaauaccuauaaugcgaacaucuucugggccaauggauaaucgugauucaacauccuuaauauuuucuuuaauuccaaaaagcaacuccuuugaaugcucauauguugaucgguauuuaggcaauuucucuaaaauaucuuuaacaacucguuuaacuaacucauacucaggccuacaaaaaauaaauaaauaaauauuguuagac     exp
    ((((((((((..(((((((...((((.((((....(((((.((((......................))))..)))))..)))))))).....)))))))..)))...((((........)))).....(((((((((......(((((..((((((((((.......))).))))))).))))))...)))))))).............))).......................)))))))....     reads  mm  sample
    ...........caagggugguuuuaccgauu...........................................................................................................................................................................................................................     5      0   LCK-3
    ....................uCuuaccgauuccccccauucc................................................................................................................................................................................................................     24     1   JT-1
    ......................uuaccgauuccccccauuU.................................................................................................................................................................................................................     12     1   JT-1
    ......................uuCccgauuccccccauucc................................................................................................................................................................................................................     1      1   LCK-2
    ......................uuNccgauuccccccauucc................................................................................................................................................................................................................     1      1   LCK-1
    ......................uuaccgauuccccccauucc................................................................................................................................................................................................................     42     0   LCK-2
    ......................uuaccgauuccccccauucc................................................................................................................................................................................................................     38     0   JT-1
    ......................uuaccgauuccccccauucc................................................................................................................................................................................................................     59     0   LCK-1
    ......................uuaccgauuccccccauuccA...............................................................................................................................................................................................................     37     1   LCK-2
    ......................uuaccgauuccccccauuccA...............................................................................................................................................................................................................     142    1   JT-1
    ......................uuaccgauuccccccauuccA...............................................................................................................................................................................................................     45     1   LCK-1
    .......................................uccccaaauaccuauaaugcgaac...........................................................................................................................................................................................     1      0   JCK-2
    ...........................................caaauaccuauGaugcgaac...........................................................................................................................................................................................     1      1   LT-3
    ..................................................................uuUugggccaauggauaaucgugauuca............................................................................................................................................................     1      1   LT-3
    .....................................................................ugggccaauggauaaucguga................................................................................................................................................................     4      0   JCK-2
    .....................................................................ugggccaauggauaaucguga................................................................................................................................................................     1      0   JCK-3
    .........................................................................ccaauggauaaucgugauucaac..........................................................................................................................................................     1      0   LT-1
```
